# Highly Potent MRI Contrast Agent Displaying Outstanding Sensitivity to Zinc Ions

**DOI:** 10.1002/anie.202014431

**Published:** 2021-01-27

**Authors:** Gaoji Wang, Goran Angelovski

**Affiliations:** ^1^ MR Neuroimaging Agents Max Planck Institute for Biological Cybernetics Tuebingen Germany; ^2^ Laboratory of Molecular and Cellular Neuroimaging International Center for Primate Brain Research (ICPBR) Center for Excellence in Brain Science and Intelligence Technology (CEBSIT) Chinese Academy of Science (CAS) Shanghai P. R. China

**Keywords:** gadolinium, magnetic resonance imaging, responsive contrast agents, zinc

## Abstract

Zinc ions play an important role in numerous crucial biological processes in the human body. The ability to image the function of Zn^2+^ would be a significant asset to biomedical research for monitoring various physiopathologies dependent on its fate. To this end, we developed a novel Gd^3+^ chelate that can selectively recognize Zn^2+^ over other abundant endogenous metal ions and alter its paramagnetic properties. More specifically, this lanthanide chelate displayed an extraordinary increase in longitudinal relaxivity (*r*_1_) of over 400 % upon interaction with Zn^2+^ at 7 T and 25 °C, which is the greatest *r*_1_ enhancement observed for any of the metal ion‐responsive Gd‐based complexes at high magnetic field. A “turn‐on” mechanism responsible for these massive changes was confirmed through NMR and luminescence lifetime studies on a ^13^C‐labeled Eu^3+^ analogue. This molecular platform represents a new momentum in developing highly suitable magnetic resonance imaging contrast agents for functional molecular imaging studies of Zn^2+^.

## Graphical abstract

A highly potent MRI contrast agent based on paramagnetic Gd^3+^ was developed to selectively recognize Zn^2+^ over other abundant endogenous metal ions. The interaction with Zn^2+^ modulates the hydration of the complex, leading to a significant increase in its longitudinal relaxivity. The new responsive platform is highly suitable for studying the Zn‐related physiological processes at high magnetic fields.

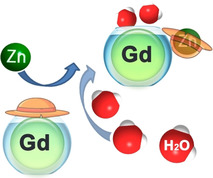

Zinc ions are found in all cells in the human body, either in free or protein‐bounded forms.^[1]^ As the second most abundant transition metal ion, Zn^2+^ plays an important role in many essential biological processes.^[2]^ For example, it is involved in numerous aspects of cellular metabolism such as the mediation of enzymes activity, the conveyance of neural signals and the transcription of genes. Both an excess and deficiency of Zn^2+^ causes different symptoms and pathologies, such as hair loss, brain or prostate cancer.^[3]^ Therefore, it is essential for healthy organs that the concentration of Zn^2+^ is perfectly balanced by transporters and metallochaperones. Magnetic resonance imaging (MRI), a non‐invasive technique with high spatial resolution, is one of the highly suitable methods for investigating the biological role of Zn^2+^ and providing early‐stage disease detection, particularly in combination with the use of contrast agents (CA).^[4]^

Application of CAs in MRI guarantees higher contrast images through the shortening of the *T*_1_ (spin‐lattice) and *T*_2_ (spin‐spin) relaxation times of the water molecule that interact with the CA.^[4a]^ Complexes of gadolinium with different polydentate chelating ligands are most frequently chosen for such purposes,[^4a^, ^5^] as they can shorten the *T*_1_ and *T*_2_ relaxation times of water protons by rapid exchange of inner‐sphere water molecules with the bulk solvent and thus enhance the MR image contrast.^[6]^ A variant of these complexes, so‐called bioresponsive or “smart” contrast agents (SCAs), are well suitable for the visualization of numerous biological processes through functional MRI (fMRI) studies, as they can alter their signal upon interaction with the desired target (e.g. metal ion of interest).[^4c^, ^7^] To this end, development of SCAs to specifically distinguish Zn^2+^ over other metal ions was initiated in the pioneering studies from Nagano and co‐workers in 2001.^[8]^ Meanwhile, many approaches and a large number of SCAs sensitive to Zn^2+^ have been reported, based mainly on Gd^3+^ complexes,[^4c^, ^9^] while the Zn‐responsive probes for other imaging modalities have also been developed.^[10]^ Recently, significant progress in performing MRI in vivo to study the function of Zn^2+^ has been made by Sherry and co‐workers. In these studies, the SCAs can only be trigged by Zn^2+^ and human serum albumin (HSA) together, resulting in a longitudinal *r*_1_ relaxivity enhancement of ≈60 % at high magnetic field (9.4 T). A greater change in *r*_1_ (≈270 %) can only be measured at low magnetic field (0.5 T).^[9a]^ Nevertheless, the former level of changes in *r*_1_ was sufficient to perform a set of important in vivo experiments to study the role of Zn^2+^ in β‐cell function and insulin release at high field.[^9a^, ^11^]

To push the limits of Zn^2+^ detection at high magnetic fields with SCAs, we approached the problem by using a structural motif we recently discovered, which shows an excellent “turn‐on” luminescence emission response to Zn^2+^.^[12]^ The SCA molecule we designed was based on the di‐(2‐picolyl)amine (DPA) as the Zn^2+^ recognition moiety, 1,4,7,10‐tetraazacyclododecane‐1,4,7‐tricarboxylic acid (DO3A) as chelator for Gd^3+^ and a tyrosine (Tyr) unit as a spacer. Herein, we protected the carboxylate of Tyr as the methyl ester, and deliberately appended an acetate moiety on the phenolic oxygen, which can act as a bridge between the DPA moiety and the DO3A chelator. Positioned in such way, the phenoxyacetic acid can interact and coordinate with either Zn^2+^ or Gd^3+^, thus playing an important role in potential alterations of relaxivity.

The preparation of the desired complex **GdL** (Figure 1) was done in analogy to the recently reported luminescence chemosensor.^[12]^ Additionally, phenoxyacetate was installed by the alkylation of the phenolic ‐OH of **1** at room temperature to smoothly provide **2** within 12 h (Scheme S1 in SI). The chelator **H_4_L** was obtained by treating **2** with TFA, followed by purification with HPLC (synthetic procedure in SI). In parallel, we prepared the chelator H_4_L*, labeled with ^13^C isotopes on the phenoxyacetate group. Here, we used 1,2‐^13^C‐*tert*‐butyl bromoacetate in the alkylation step, instead of the molecule with normal isotope abundance. The final complexes **GdL**, **TbL** or EuL* were prepared by treating **H_4_L**/H_4_L* with the respective metal ion salt.

**Figure 1:**
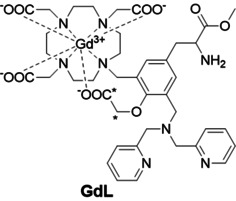
Chemical structure of **GdL**. Asterisks show the positions labeled with ^13^C isotopes in the respective EuL* complex.

To evaluate the response of **GdL** toward Zn^2+^ and its potential as a MRI contrast agent, a series of ^1^H NMR relaxometric titrations were performed at 7 T and 25 or 37 °C. Both *r*_1_ and *r*_2_ were measured after every addition of Zn^2+^, resulting in an unprecedented change in *r*_1_ relaxivity from 2.05 to 10.30 mM^−1^ s^−1^ (≈400 %) at 25 °C and 1.51 to 6.04 mM^−1^ s^−1^ (≈300 %) at 37 °C upon saturation with Zn^2+^ (Figure 2 a and S1a in SI). Such a significant increase in *r*_1_ upon the addition of 1 equiv of Zn^2+^ is, to the best of our knowledge, the highest change reported thus far for a metal cation‐sensitive Gd‐based CA operating at the high magnetic fields. These observations seemingly indicate substantial changes in the coordination geometry around the Gd^3+^ center that lead to changes in the inner‐sphere hydration (vide infra). Indeed, when the HEPES buffer was exchanged for PBS, the overall *r*_1_ increased from 1.82 to 7.37 mM^−1^ s^−1^ (≈300 %) and 1.47 to 5.58 mM^−1^ s^−1^ (≈280 %) for 25 and 37 °C, respectively. These indicated still an outstanding *r*_1_ response, however slightly affected by the formation of ternary complexes between the phosphates and Gd^3+^.^[13]^ The changes in *r*_2_ values in both buffered media followed similar trends (Figure S1 in SI).

**Figure 2:**
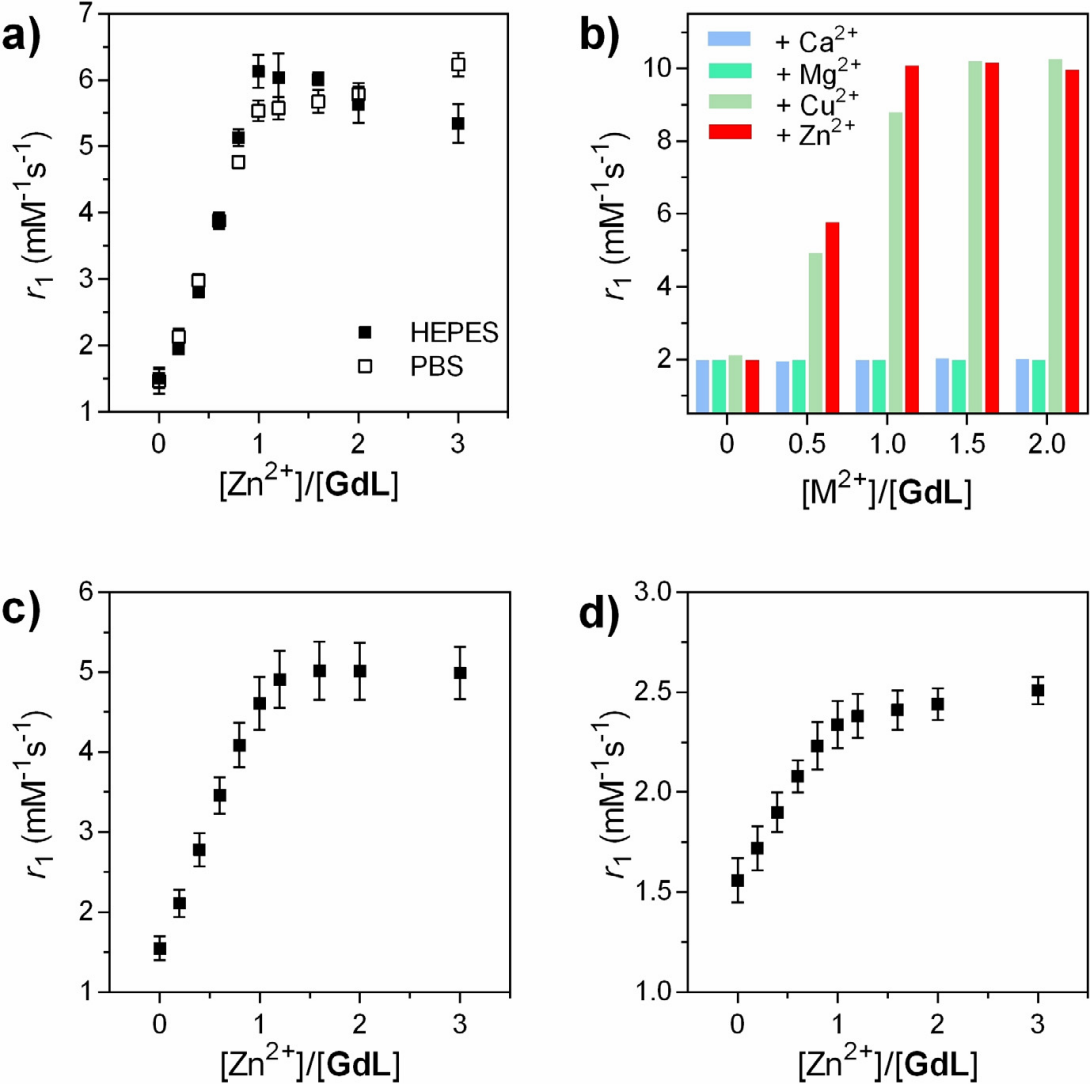
Longitudinal relaxivity of **GdL** at 7 T. a) *r*_1_ in the presence of various concentrations of ZnCl_2_ in HEPES (50 mM) or PBS (50 mM) ([**GdL**]=3 mM, pH 7.4 and 37 °C); b) *r*_1_ in the presence of different quantities of Ca^2+^, Mg^2+^, Cu^2+^ or Zn^2+^ ([**GdL**]=3 mM, 50 mM HEPES, pH 7.4 and 25 °C). c,d) *r*_1_ in the presence of various concentrations of ZnCl_2_ in c) HSA (0.6 mM) or d) human serum (both [**GdL**]=1 mM, pH 7.4 and 37 °C). Values in (a), (c) and (d) represent the mean and standard deviation from 3 independent measurements.

The selectivity of **GdL** towards Zn^2+^ was tested in separate experiments with metal ions commonly present in biological systems, such as Ca^2+^, Mg^2+^ and Cu^2+^ (Figure 2 b). No obvious response of **GdL** toward any other cation was observed, with the exception of Cu^2+^. However, such potential competition can be omitted because the concentration of free Cu^2+^ in living cells is very low.^[14]^

Finally, the relaxometric behavior of **GdL** was probed in more complex environments at physiological pH and 37 °C. The *r*_1_ enhancement in the presence of HSA (0.6 mM in PBS) exceeded 200 % upon saturation with ZnCl_2_ (Figure 2 c). The *r*_1_ and *r*_2_ titrations of HSA with **GdL** or **GdLZn** complex showed that interaction of **GdL**/**GdLZn** with the protein is not pronounced and is slightly notable only in the sub‐equimolar amounts; however, the bicarbonates (25 mM) have remarkable influence on both *r*_1_ and *r*_2_ values, as expected (Figure S1b,c in SI). Nevertheless, the titration of **GdL** with ZnCl_2_ in human serum resulted in the increase in *r*_1_ of around 60 % (Figure 2 d), which is the highest change observed so far for any of the Zn‐sensitive SCA.^[9a]^

The specificity toward Zn^2+^ and its strong relaxivity enhancement, suggest that **GdL** could be a highly promising molecular probe for the imaging of this biomarker. We therefore performed additional characterization of the complex to assess its coordination properties and estimate its potential for MRI applications. The binding affinity of **GdL** towards Zn^2+^ was determined by means of isothermal titration calorimetry in both HEPES and PBS (Figure S2 in SI). The obtained dissociation constant resulted in the values *K*_d(GdZnL)_=543±39 and 552±76 nM in HEPES and PBS, respectively. This is in line with the values we obtained for the luminescence sensor with a similar structure;^[12]^ also, the results indicate that the binding affinity is not affected by the ternary complex formation between the phosphates and Gd^3+^ (vide supra).

The stability of **GdL** was investigated in a transmetallation experiment against Zn^2+^, a major potential competitor for the displacement of Gd^3+^.^[15]^ For this, **GdL** was exposed to 2 equiv of Zn^2+^ in a phosphate buffer (50 mM, pH 7.0) at 25 and 37 °C. The replacement of Gd^3+^ for Zn^2+^ was monitored by measuring the relaxation rate of the solution over a period of 120 h (Figure S3 in SI). Subsequently, a “thermodynamic index” was calculated as the ratio of the paramagnetic relaxation rate after a given period, compared with the starting value. For **GdL**, this index resulted in values 90, 81 and 75 % after 24, 72 and 120 h for the temperature 37 °C, respectively, indicating high stability of the investigated SCA.

To confirm the binding pattern of Zn^2+^ with **GdL**, an analogous Eu^3+^ complex EuL* was prepared with the ^13^C‐labeled phenoxyacetate group (vide supra). Thereafter, a series of ^13^C NMR spectra of 15 mM EuL* were recorded with increasing concentrations of ZnCl_2_ (Figure 3 a and S4 in SI), in analogy to the experiments previously conducted by Meade and co‐workers.^[16]^ In the absence of Zn^2+^, the spectrum showed two broad signals at 93.5 ppm and 211.7 ppm. Once Zn^2+^ was gradually added to the sample, these signals slowly disappeared, while two sharp doublets at 71.7 ppm and 175.6 ppm appeared, owing to the coupling interactions with the neighboring isotopic carbon atom. After the addition of one equiv of Zn^2+^, the broader signals at 93.5 ppm and 211.7 ppm disappeared completely. Furthermore, the doublets at 71.7 ppm and 175.6 ppm experienced a small shift with further additions of Zn^2+^, indicating the formation of the second type of species in the excess of Zn^2+^; no further changes in ^13^C NMR spectra were observed beyond the second equiv of Zn^2+^.

**Figure 3:**
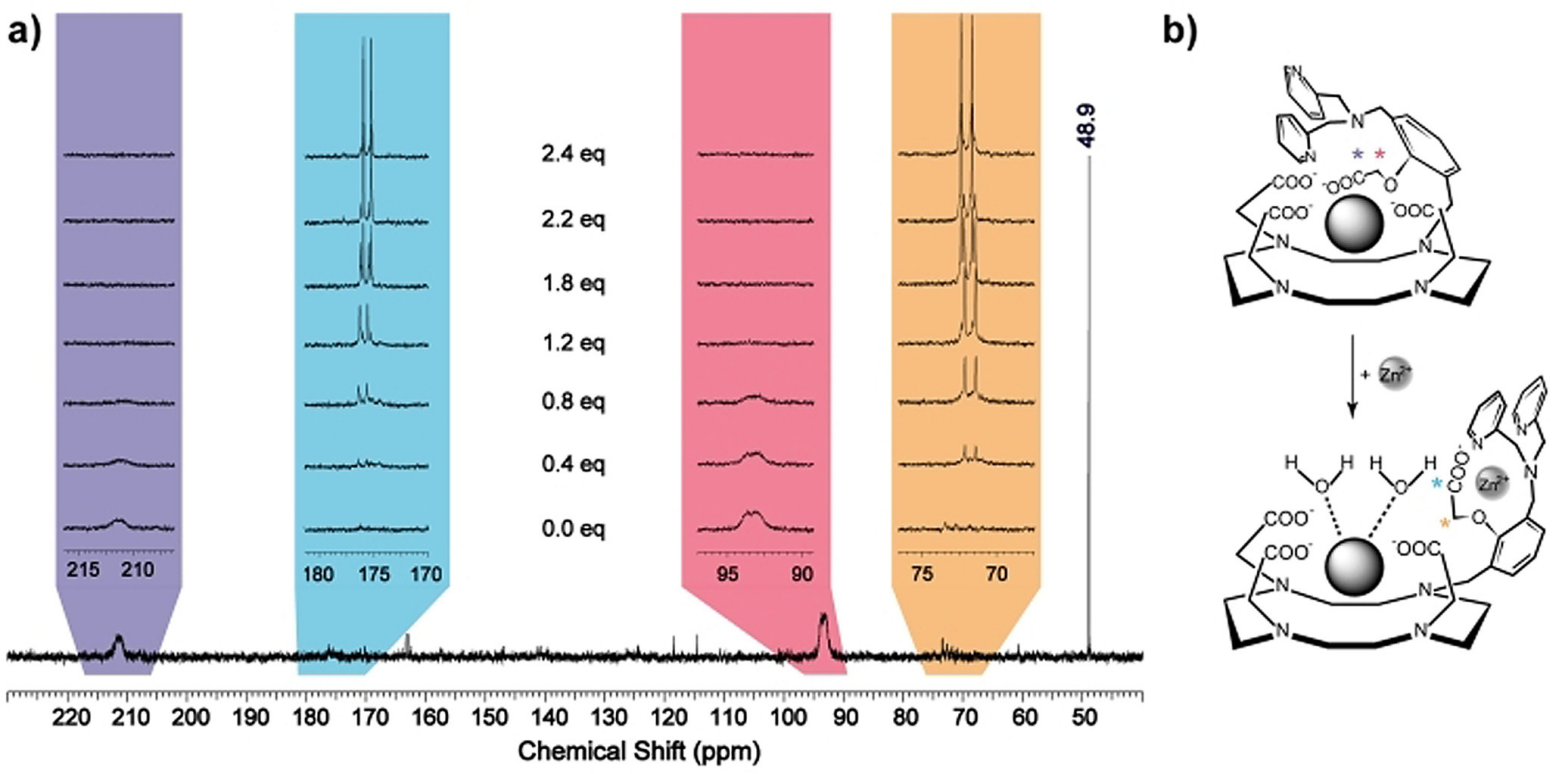
a) ^13^C NMR spectra of EuL* (15 mM) in the presence of 0–2.4 equiv of Zn^2+^ at 25 °C and 75 MHz (note: 48.9 ppm is the referent signal of ^13^CH_3_OH). b) Proposed interaction of the phenoxyacetate group with the paramagnetic metal center (top) and Zn^2+^ (bottom), which leads to an increase in hydration number and the “turn‐on” response.

This specific experiment provided the strongest ground for the mechanism responsible for changes in the coordination around the paramagnetic metal ion that are caused by Zn^2+^. Namely, broad and shifted signals in the Zn‐free state indicated coordination of phenoxyacetate group to the paramagnetic Eu^3+^. Moreover, the amplitude of the shifts (≈36 and ≈22 ppm for the carboxylate and methylene group, respectively), suggests the carboxylate being much closer to Eu^3+^, hence the larger shift. Upon addition of Zn^2+^, the phenoxyacetate group flips from Eu^3+^ to Zn^2+^, forming a Zn^2+^ complex with the DPA moiety (Figure 3 b). Consequently, the ^13^C signals of the carbons in the phenylacetate recover in intensity, multiplicity and the usual frequency shift in ^13^C NMR for the respective functional groups. Moreover, the minor change in the shift of doublets between 1–2 equiv of Zn^2+^ indicates that EuL*:Zn^2+^ stoichiometry likely moves from 1:1 to 1:2 complex formation, which was also observed in the case of the luminescent phenoxy analogue.^[12]^ However, it is obvious that, irrespective of the type of formed species (1:1 or 1:2), already the first equiv of Zn^2+^ causes the decoordination of the phenoxyacetate from the paramagnetic metal center. We note that ^1^H NMR of EuL* at 25 °C displays sharp resonances, suggesting the existence of only one of the isomers, namely the twisted antisquare prismatic species (TSAP, Figure S5 in SI).

The coordination features of this system were also studied by means of the luminescence lifetime experiments. For this purpose, the luminescence lifetimes of EuL* and **TbL** in D_2_O or H_2_O with and without Zn^2+^ were measured at pH 7.4 and 25 °C. Subsequently, the number of the water molecules coordinated to the Eu^3+^/Tb^3+^ center (*q*) was estimated (Table 1).

**Table 1:** Luminescence lifetimes of the EuL* (5 mM) and **TbL** (1 mM) in H_2_O and D_2_O with and without Zn^2+^, and the calculated *q* values.

	**LnL** only			**LnL** + Zn^2+^ (2 equiv)			
	*τ* ${}_{H{}_{2}O}$ [ms]	*τ* ${}_{D{}_{2}O}$ [ms]	*q*	*τ* ${}_{H{}_{2}O}$ [ms]	*τ* ${}_{D{}_{2}O}$ [ms]	*q*	Δ*q*
EuL^*^	0.89	1.25	0.1	0.42	1.02	1.4	1.3
**TbL**	1.91	1.96	0.0	1.30	2.37	1.5	1.5

In the absence of Zn^2+^, both EuL* or **TbL** are non‐hydrated, explaining the very low initial *r*_1_ value of **GdL** (vide supra). Upon Zn^2+^ addition, the estimated *q* values are 1.4 and 1.5 for EuL* and **TbL**, respectively. In parallel, the *r*_1_ value of **GdL** upon Zn^2+^ binding dramatically increases, which matches this observation. These results confirm the mechanism that assumes complete coordination of the Gd^3+^ with DO3A and DPA units, leading to *q*=0 in the absence of Zn^2+^. Once Zn^2+^ is added, the whole DPA unit including the phenoxyacetate is involved in coordination with Zn^2+^, giving rise to higher hydration of the paramagnetic chelate and therefore the boost in *r*_1_ (Figure 3 b). Additionally, this coordination probably causes a concurrent decrease in *τ*_R_ with an increase in the outer‐sphere hydration, which contribute to the overall *r*_1_, as previously observed on structurally similar systems that are Ca^2+^ sensitive. These also exhibited high *r*_1_ values, while being monohydrated complexes.^[17]^

The potential of this complex to serve as a *T*_1_‐weighted SCA was demonstrated in vitro in an MRI experiment on tube phantoms. Six tubes containing **GdL** alone or with added Zn^2+^, Mg^2+^ or Ca^2+^ were imaged in a 7 T MRI scanner. The results indicated a great enhancement in the MR signal intensity for tubes with **GdL** and 0.5 or 1.0 equiv of Zn^2+^, whereas no obvious difference was observed in tubes where Ca^2+^ or Mg^2+^ were added (Figure 4 and Table S1 in SI). The collected MR data also confirmed that a selective “turn‐on” response of **GdL** can be visualized in a Zn‐rich environment.

**Figure 4:**
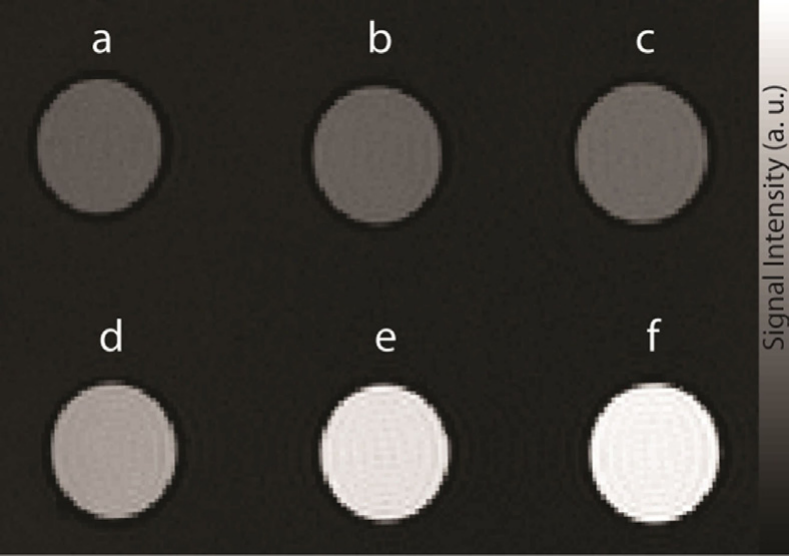
*T*_1_‐weighted MR images of tube phantoms at 7 T of a 1 mM solution of **GdL** in 50 mM HEPES buffer (pH 7.4 and ≈22 °C). The tubes were positioned in the following order: a) **GdL** only, b)+1.0 equiv Mg^2+^, c)+1.0 equiv Ca^2+^, d)+0.5 equiv Zn^2+^, e)+1.0 equiv Zn^2+^, f)+2.0 equiv Zn^2+^.

In summary, we report a novel paramagnetic complex appended with DPA as a Zn^2+^ recognition moiety and phenoxyacetate as a trigger for the “turn‐on” relaxometric response. The overall *r*_1_ relaxivity enhancement reached 400 % upon Zn^2+^ addition, which is, to the best of our knowledge, the highest *r*_1_ change observed to date for this type of ion‐sensitive SCA at high magnetic fields. The additional experiments demonstrated high binding affinity and specificity of the complex toward Zn^2+^ and confirmed the existence of the “turn‐on” mechanism. Indeed, this system displays the most desirable properties for a SCA, which encompass high *q* modulation, followed by a massive increase in relaxivity. These features are highly preferred for the development of potent probes for the molecular imaging of biomarkers. With the new paramagnetic system presented in this work, the field of functional imaging of Zn^2+^ is receiving an important tool to enable substantial and faster progress.

## Conflict of interest

The authors declare no conflict of interest.

## Supporting information

As a service to our authors and readers, this journal provides supporting information supplied by the authors. Such materials are peer reviewed and may be re‐organized for online delivery, but are not copy‐edited or typeset. Technical support issues arising from supporting information (other than missing files) should be addressed to the authors.

Supplementary
