# Supplementary material for: Highly Potent MRI Contrast Agent Displaying Outstanding Sensitivity to Zinc Ions
Source: Angew Chem Int Ed Engl. 2021 Jan 27;60(11):5734–8. doi: 10.1002/anie.202014431 (PMC7986093; doi:10.1002/anie.202014431)
Supplement: Supplementary file 1 — Supplementary [file ANIE-60-5734-s001.pdf]

Angewandte Chemie
Eine Zeitschrift der Gesellschaft Deutscher Chemiker

Supporting Information

Highly Potent MRI Contrast Agent Displaying Outstanding Sensitivity to Zinc Ions

*Gaoji Wang and Goran Angelovski**

anie_202014431_sm_miscellaneous_information.pdf

Materials and instrumentation

The reagents were purchased from commercial sources and were used without further purification. Albumin from human serum and the human serum were purchased from Sigma-Aldrich Chemie GmbH, Germany (catalogue numbers A3782 and P2918, respectively). Compound **1** was synthesized following a previously published procedure.[1] Purification of synthesized compounds was performed using silica gel 60 (0.03–0.2 mm) from Carl Roth (Germany). The final ligand and metallated complexes were purified using preparative HPLC on a Varian PrepStar system equipped with the UV−vis detector model 335 and a binary pump model SD-1 manual injector, controlled by Star chromatography workstation version 6.3 software. All fluorescence spectra were recorded on a QuantaMaster™ 3 PH fluorescence spectrometer from Photon Technology International, Inc. (USA). Low resolution mass spectra were recorded on an ion trap SL 1100 system Agilent with an electrospray ionization source. High resolution mass spectra were recorded on a Bruker Daltonics APEX II (FT-ICR-MS) with an electrospray ionization source. ^{1}H and ^{13}C NMR spectra were recorded on a Bruker Avance III 300 MHz spectrometer at 25 °C. Processing was performed using TopSpin 2.1 (Bruker GmbH) and ACD/SpecManager 9.0 (Advanced Chemistry Development, Inc.). The NMR spectra were recorded using either $CDCl_3$ or D_2O and referenced to TMS/TSP. The concentration of Eu^{3+}, Gd^{3+} and Tb^{3+} in analyzed solutions were determined using the bulk magnetic susceptibility shift (BMS).[2] ^{1}H NMR relaxometric titrations were performed on the same instrument (Bruker Avance III 300 MHz spectrometer) at 25 or 37 °C. Isothermal titration calorimeter (ITC) experiments were performed on the MicroCal PEAQ-ITC (MicroCal™, Malvern Panalytical, UK). MRI experiments were performed on Bruker BioSpec 70/30 USR magnet (software version Paravision 5.1) using Bruker volume coil (RF RES 300 1H 075/040 QSN TR).

Synthetic procedures

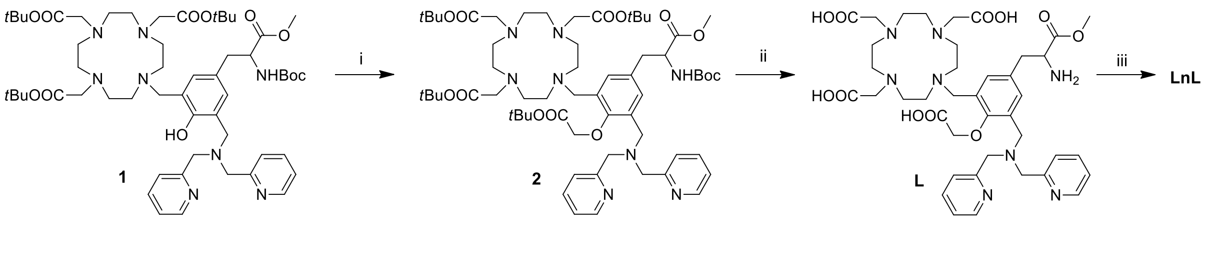

Scheme S1. The synthetic route for complexes **GdL** and **TbL**. Reagents and conditions: i) $BrCH_2COO\textit{t}Bu$, K_2CO_3, KI, MeCN, R.T., 12 h; ii) TFA, CH_2Cl_2, R.T., 6h; iii) $LnCl_3{\cdot}6H_2O$ (Ln = Tb^{3+}, Gd^{3+}), H_2O, 12 h.

3-[3-[(bis-pyridin-2-ylmethyl-amino)-methyl]-4-*tert*-butoxycarbonylmethoxy-5-(4,7,10-tris-*tert*-butoxycarbonylmethyl-1,4,7,10tetraaza-cyclododec-1-ylmethyl)-phenyl]-2-*tert*-butoxycarbonylamino-propionic acid methyl ester (2).

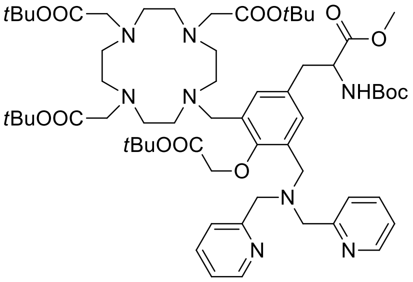

Compound **1** (0.517 g, 0.50 mmol) was added to a suspension of K_2CO_3 (0.138 g, 1.00 mmol) and KI (0.166 g, 1.00 mmol) in MeCN (3 mL). The mixture was stirred 10 min at room temperature. Then, *tert*-butyl bromoacetate (0.146 g, 0.75 mmol) was added to the reaction mixture, followed by stirring for 12 h at room temperature. Upon reaction completion, the reaction mixture was filtered and the filtrate was evaporated under vacuum. The crude residue was purified by silica gel column chromatography using CH_2Cl_2/MeOH (v/v, 20:1) as the eluent, affording 0.465 g (81%) of compound **2** as a light yellow oil. **^{1}H NMR** ($CDCl_3$, 300 MHz): δ (ppm): 1.09–1.55 (m, 45H, CCH_3); 2.08–3.25 (br, 24H, NCH_2); 3.34, 3.35, 3.36 (s, 3H, OCH_3); 3.76, 3.63 (s, 8H, NCH_2C); 4.29 (s, 2H, CCH_2O); 4.35–4.57 (m, 1H, NHC*H*); 5.70–5.98, 7.21–7.35 (br, 2H, phenolic); 7.03–7.20 (br, 2H, NCHC*H*), 7.36–7.56 (br, 2H, NCC*H*), 7.57–7.76 (br, 2H, CCHC*H*), 8.37–8.55 (br, 2H, NC*H*CH). **^{13}C NMR** ($CDCl_3$, 75 MHz): δ (ppm): 26.5–29.1 (15C, CCH_3); 37.3 (1C, phCH_2CH); 48.7, 49.2, 49.5, 51.9, 52.9, 55.9, 56.0, (12C); 57.0 (1C, NH_2*C*H); 59.4 (2C, pyCH_2); 71.3 (1C, C=OCH_2O); 79.1; 82.0, 82.3 (5C, *C*CH_3); 121.9, 122.2, 122.6, 123.3 (4C, CCO*C*, NCH*C*H); 128.8 (C, CH_2*C*CH); 131.2, 131.8 (2C, C*C*HC); 132.8 (1C, CH*C*CH); 136.2, 136.9 (2C, CCH*C*H); 148.4, 148.8 (2C, N*C*HCH); 151.5 (1C, CH_2O*C*); 155.1 (1C, NH*C*O); 158.4 (2C, N*C*CH); 167.7 (1C, *C*=OCH_2O); 172.1, 172.3, 173.2 (4C, *C*O). ESI-TOF/MS: (*m/z*) $[M+H]^+$ calcd. for $C_{61}H_{95}N_8O_{13}^+$: 1147.7013; found: 1147.7000.

2-amino-3-[3-[(bis-pyridin-2-ylmethyl-amino)-methyl]-4-carboxymethoxy-5-(4,7,10-tris-carboxymethyl-1,4,7,10tetraaza-cyclododec-1-ylmethyl)-phenyl]- propionic acid methyl ester (L).

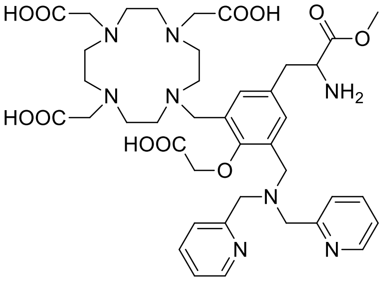

Compound **2** (0.275 g, 0.24 mmol) was dissolved in TFA/CH_2Cl_2 (2 mL, v/v 80/20) and the solution was stirred for 6 hours at room temperature. The reaction mixture was evaporated to

dryness and purified by HPLC using MeCN/H_2O as the eluent, affording 0.182 g (76%) of compound **L** as a light yellow oil. **^{1}H NMR** (D_2O, 300 MHz): δ (ppm): 2.69–3.54 (br, 24H, C*H*$_2$); 3.62–3.91 (br, 7H, C*H*$_3$, C*H*$_2$); 4.08 (s, 2H, ArO-C*H*$_2$); 4.29–4.46 (br, 5H, C*H*NH$_2$, C*H*$_2$); 7.05–7.23, 7.34–7.58, 7.76–8.00, 8.39–8.66 (br, 10H, Ar*H*). **^{13}C NMR** (D_2O, 75 MHz): δ (ppm): 32.6 (1C, Ar*C*H$_2$CH); 45.7, 47.3 (2C, Ar*C*H$_2$N); 46.2 (1C, *C*H$_3$); 46.6 (1C, *C*HNH$_2$); 48.2, 48.4, 49.5, 50.0, 51.6, 53.5, 53.8, 54.2, 54.4 (11C, N*C*H$_2$CH$_2$, N*C*H$_2$CO); 56.0 (2C, Py*C*H$_2$N); 71.3 (1C, ArO*C*H$_2$); 124.0, 130.0, 130.5, 131.4, 132.1 (5C, Ar); 123.2, 123.9, 138.3, 145.9, 149.1 (10C, Py); 154.1 (1C, *C*-OCH$_2$), 166.8, 168.0, 168.6, 173.8, 176.4 (5C, *C*O). ESI-TOF/MS: (*m/z*) $[M+H]^+$ calcd. for $C_{40}H_{55}N_8O_{11}^+$: 823.3985; found: 823.3973.

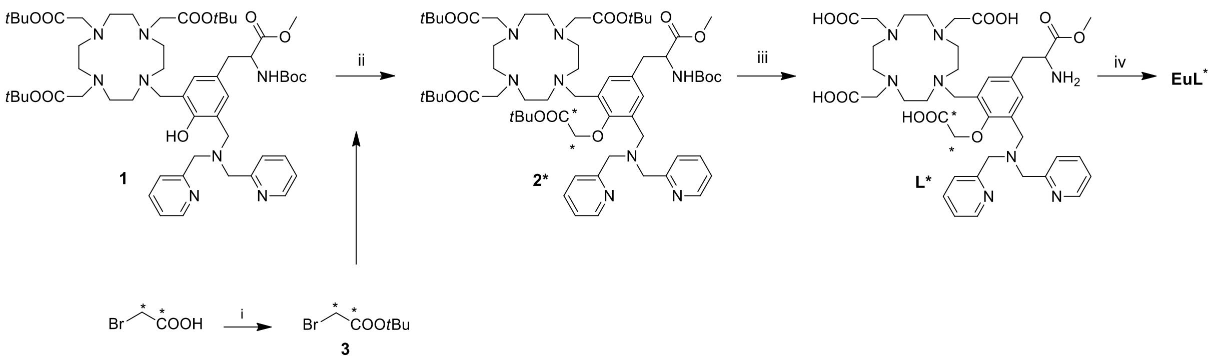

Scheme S2. The synthetic route for complex **EuL***. Reagents and conditions: i) H_2SO_4, $MgSO_4$, *t*BuOH, DCM, R.T., 15 h. ii) **3**, K_2CO_3, KI, MeCN, R.T., 12 h; iii) TFA, CH_2Cl_2, R.T., 6 h; iv) $EuCl_3$•6H_2O, H_2O, 12 h.

1,2-^{13}C-*tert*-Butyl bromoacetate (3)

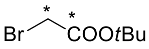

Concentrated H_2SO_4 (0.19 mL) was added slowly to a vigorously stirred suspension of $MgSO_4$ (1.300 g, 10.80 mmol) in CH_2Cl_2 (7 mL) The mixture was stirred for 20 minutes, after which the isotopically labeled 1,2-^{13}C-bromoacetic acid (0.507 g, 3.60 mmol) was added, followed by addition of *t*BuOH (1.5 mL). The mixture was stirred for 15 h at room temperature. The insoluble matter was removed by vacuum filtration. The filtrate was transferred to a separatory funnel and washed with water (10 mL) and saturated sodium bicarbonate (10 mL). The aqueous layer was extracted with CH_2Cl_2 (3× 3 mL). The combined organic layers were washed with brine and dried over anhydrous $MgSO_4$. The solvent was evaporated under reduced pressure to afford **3** as a light-yellow liquid (0.341 g, 48%). **^{1}H NMR** ($CDCl_3$, 300 MHz): δ (ppm): 1.48 (s, 9H, CC*H*$_3$); 3.49, 3.50, 3.99, 4.01 (d, *J*= 4.5 Hz, 2H, BrC*H*$_2$). **^{13}C NMR** ($CDCl_3$, 75 MHz): δ (ppm): 25.6–30.2 (4C, C*C*H$_3$, Br*C*); 82.8 (1C, *C*CH$_3$); 165.8, 166.6 (1C, *C*=O).

^{13}C-labeled 3-[3-[(Bis-pyridin-2-ylmethyl-amino)-methyl]-4-*tert*-butoxycarbonylmethoxy-5-(4,7,10-tris-*tert*-butoxycarbonylmethyl-1,4,7,10tetraaza-cyclododec-1-ylmethyl)-phenyl]-2-*tert*-butoxycarbonylamino-propionic acid methyl ester (2*).

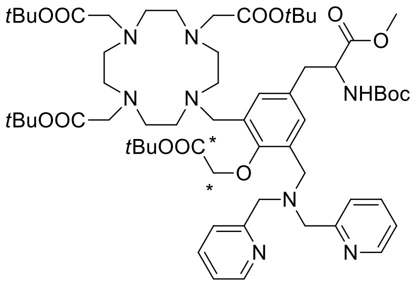

Procedure was the same as for compound **2**. Starting materials: compound **1** (0.238 g, 0.23 mmol), K_2CO_3 (0.064 g, 0.46 mmol), KI (0.076 g, 0.46 mmol) and compound **3** (0.054 g, 0.276 mmol). Yield: 0.185 g (70%) of compound **2*** as a light yellow oil. **^{1}H NMR** ($CDCl_3$, 300 MHz): δ (ppm): 1.20–1.55 (m, 45H, CCH_3); 2.22–3.33 (br, 24H, NCH_2); 3.44 (s, 3H, OCH_3); 3.62–3.94 (s, 10H, NCH_2C); 4.02–4.15, 4.39–4.54 (s, 2H, CCH_2O); 4.54–4.63 (m, 1H, NHC*H*); 6.92–7.31 (br, 2H, NCHC*H*; 1H, phenolic), 7.38–7.63 (br, 2H, NCC*H*; 1H, phenolic), 7.64–7.82 (br, 2H, CCHC*H*), 8.48–8.71 (br, 2H, NC*H*CH). **^{13}C NMR** ($CDCl_3$, 75 MHz): δ (ppm):): 27.7, 27.8, 28.0, 28.2 (15C, C*C*H_3); 37.6 (1C, ph*C*H_2CH); 48.9–59.5 (15C); 70.7, 71.0, 71.9, 72.1 (1C, C=O^{13}*C*H_2O); 79.3; 82.2, 82.5 (5C, *C*CH_3); 122.1 (2C, NCH*C*H); 123.0 (2C, NC*C*H); 128.3, 128.9 (2C, C*C*HC); 131.1–133.2 (3C, ph*C*CH_2); 136.5 (2C, CCH*C*H); 148.8 (2C, N*C*HCH); 151.6 (1C, CH_2O*C*); 155.0, 155.4, 155.8 (1C, NH*C*O); 158.6 (2C, N*C*CH); 167.3, 167.98, 168.2, 168.6 (1C, ^{13}*C*=OCH_2O); 172.4, 173.3 (4C, *C*O). ESI-TOF/MS: (*m/z*) $[M+H]^+$ calcd. for $^{13}C_2C_{59}H_{95}N_8O_{13}^+$: 1149.7080; found: 1149.7063.

^{13}C-labeled 2-amino-3-[3-[(bis-pyridin-2-ylmethyl-amino)-methyl]-4-carboxymethyloxy-5-(4,7,10-tris-carboxymethyl-1,4,7,10tetraaza-cyclododec-1-ylmethyl)-phenyl]-propionic acid methyl ester (L*).

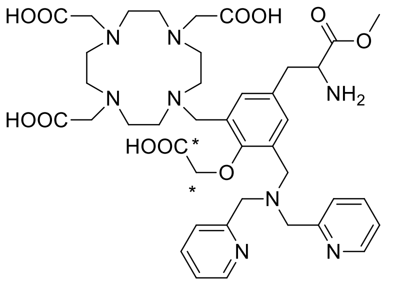

Procedure was the same as for compound **L**. The starting material compound **2*** (0.185 g, 0.161 mmol) afforded 0.090 g (68%) of compound **L*** as a light yellow oil. **^{1}H NMR** (D_2O, 300 MHz): δ (ppm): 2.96–3.60 (br, 24H, NCH_2); 3.63 (s, 3H, OCH_3); 3.85, 4.04, 4.11, 4.17, 4.22 (s, 8H, NCH_2C; m, 1H, NH_2C*H*); 7.39 (s, 2H, phenolic); 7.81–7.95 (br, 2H, NCC*H*); 7.96–8.10 (br, 2H, CCHC*H*); 8.38–8.50 (br, 2H, CCHC*H*); 8.59–8.70 (br, 2H, NC*H*CH). **^{13}C NMR** (D_2O, 75 MHz): δ (ppm): 34.8 (1C, ph*C*H_2CH); 48.0–50.1, 52.5, 53.6, 54.9, 58.8, 59.6,

65.9 (15C); 70.6, 71.4 (1C, C=O^{13}*C*H_2O); 126.5, 127.1 (4C, CCO*C*, NCH*C*H); 130.1 (C, CH_2*C*CH); 132.5, 133.5 (2C, C*C*HC); 134.5 (1C, CH*C*CH); 141.4 (2C, CCH*C*H); 147.4 (2C, N*C*HCH); 152.2 (1C, CH_2O*C*); 155.8 (1C, NH*C*O); 169.3 (2C, N*C*CH) ; 170.1 (4C, *C*O); 171.1, 172.0 (1C, ^{13}*C*=OCH_2O). ESI-TOF/MS: (*m/z*) $[M+H]^+$ calcd. for $^{13}C_2C_{38}H_{55}N_8O_{11}{}^+$: 825.4052; found: 825.4054.

General procedure for the preparation of the Eu^{3+}, Gd^{3+} and Tb^{3+} complexes. The introduction of the gadolinium, terbium (for **L**) or europium (for **L***) ions into the macrocyclic framework was carried out at pH ~7.0 adjusted by 0.1 M NaOH solution. To a stirred aqueous solution of ligand, a solution of $EuCl_3$•6H_2O, $GdCl_3$•6H_2O or $TbCl_3$•6H_2O was prepared in water and was added dropwise to the ligand solution in 1:1 molar ratios. The pH of the solution was periodically adjusted to 7.0 by addition of 0.1 M NaOH solution. The reaction mixture was stirred at room temperature for 12 h then purified by HPLC, respectively. The yellow solid compound was obtained by lyophilization. The formation of the metal complexes **TbL**, **GdL** and **EuL*** were confirmed by mass spectrometry.

GdL. ESI- TOF/MS: (*m/z*) $[M]^-$ calcd. for $C_{40}H_{50}GdN_8O_{11}{}^-$: 976.2846; found: 976.2866.

EuL*. ESI-TOF/MS: (*m/z*) $[M+H]^+$ calcd. for $^{13}C_2C_{38}H_{52}EuN_8O_{11}{}^+$: 975.3040; found: 975.3050.

TbL. ESI-LRMS: (*m/z*) $[M]^-$ calcd. for $C_{40}H_{50}TbN_8O_{11}{}^-$: 977.3; found: 977.3.

NMR measurements

Relaxometric titrations: Proton longitudinal relaxometric titrations with Zn^{2+} were performed at 7 T, pH 7.4 (50 mM HEPES buffer) and 25 °C, using using inversion recovery (T_1) and Car–Purcell-Meiboom-Gill (T_2) pulse sequences. A $ZnCl_2$ solution of known concentration was added stepwise to the **GdL** solution (starting concentration 1.0 or 3.0 mM Gd^{3+}), and measurements of T_i (i=1, 2) were performed after each addition of the analyte. The longitudinal and transverse relaxivities were calculated from Eq. S1 where $T_{i,\mathrm{obs}}$ is the measured T_i, $T_\mathrm{i,d}$ is the diamagnetic contribution of the solvent, and [Gd] is the actual Gd^{3+} concentration at each point of the titration.

$$1/T_\mathrm{i,obs} = T_\mathrm{i,d} + r_i \times [\mathrm{Gd}],\ \mathrm{i}=1, 2 \qquad \text{Eq. S1}$$

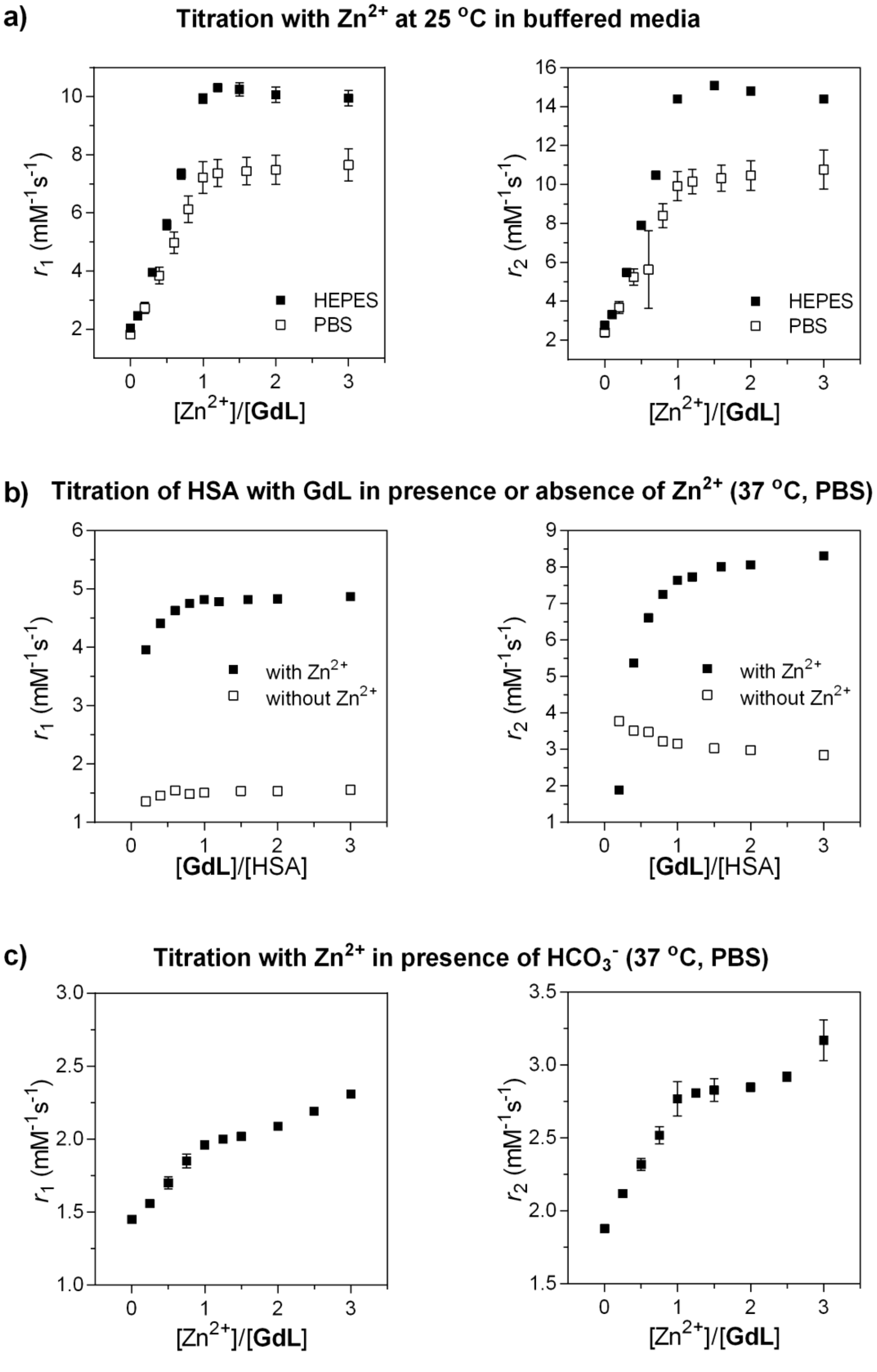

Figure S1. Longitudinal and transverse relaxivities of **GdL** at 7 T in the presence of various concentrations of $ZnCl_2$ in HEPES (50 mM) or PBS (50 mM) (pH 7.4 and 25 or 37 °C). Concentrations of **GdL** used: a) 3 mM, b-c) 1 mM.

Metal ion selectivity: For metal ion selectivity experiments, stock solutions (50 mM) of $CaCl_2$, $MgCl_2$, $CuCl_2$ and $ZnCl_2$ were prepared. The samples of **GdL** (3 mM) were prepared by the dilution method using HPLC grade water and HEPES buffer (50 mM, pH 7.4). The longitudinal relaxivities were performed in same way as described above after addition of 0, 0.5, 1.0, 1.5 and 2.0 equiv. of the respective metal ions to the solution of **GdL**.

ITC experiments

The experiments were carried out by placing **GdL** (50 μM) in the reaction cell and $ZnCl_2$ (300 μM) in the syringe. All data were recorded in HEPES or PBS (50 mM) at pH 7.4.

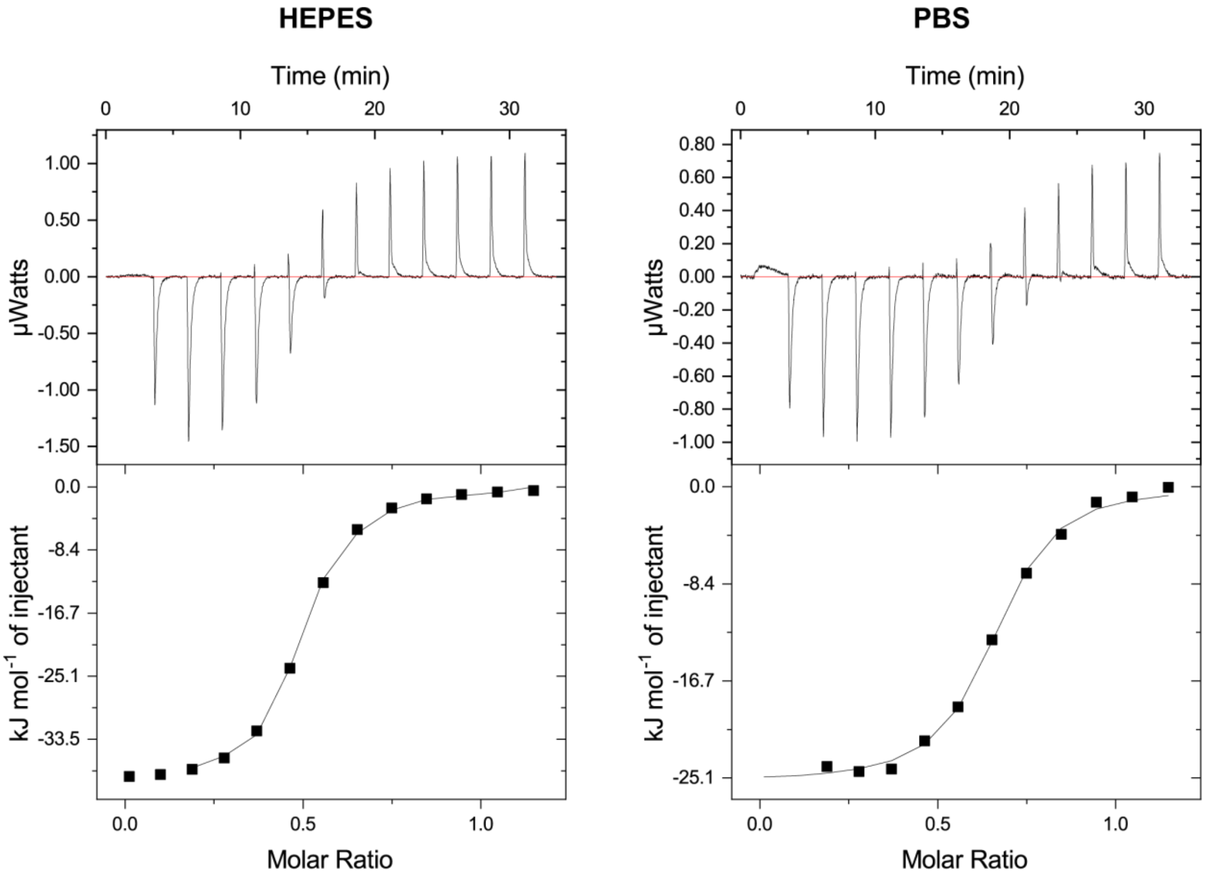

Figure S2. The raw thermogram (top) and the binding isotherm (bottom) obtained in the ITC experiment of **GdL** with Zn^{2+} in HEPES (left) and PBS (right).

Transmetalation study

The transmetalation with Zn^{2+} was performed in 50 mM phosphate buffer pH 7.0 at 25 °C. Stock solutions of the **GdL** (27 mM), and zinc chloride (250 mM) were prepared. Exact concentration of [Gd] was determined by BMS method. Calculated volumes were pipetted with calibrated pipettes into small glass vials to obtain these concentrations: 50 mM PBS, 3 mM **GdL**, 6 mM $ZnCl_2$. Deionized water was used to complete the volume to 350 μL. Thereafter, the longitudinal relaxation times were measured periodically over the period of 3 days.

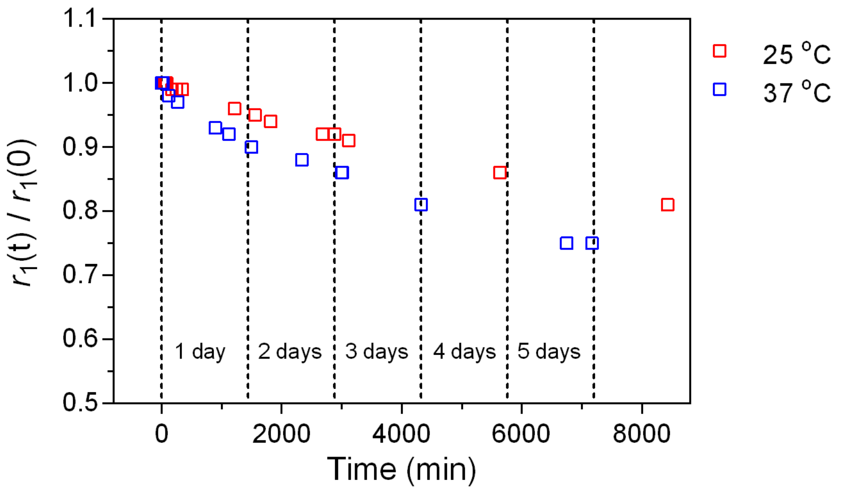

Figure S3. Relaxivity rate variation of **GdL** (3 mM) with Zn^{2+} versus time. All data were recorded in 50 mM PBS buffer at pH 7.0**.**

$^{1}H/^{13}C$ NMR spectroscopy of ^{13}C-labeled EuL*

^{13}C NMR spectra were measured with 15 mM **EuL*** in D_2O with 0 to 2.4 equiv. of Zn^{2+} (50 mM HEPES buffer, pH 7.4, 25 °C). A capillary filled with ^{13}C-labeled methanol was placed into the **EuL*** solution as an external reference. All measurements were performed under the same NMR parameters (receiver gain, number of scans). Twelve ^{13}C NMR experiments were performed by adding the following amount of Zn^{2+} (see Figure S4 top): 1: 0 equiv., 2: 0.2 equiv., 3: 0.4 equiv., 4: 0.6 equiv., 5: 0.8 equiv., 6: 1.0 equiv., 7: 1.2 equiv., 8: 1.4 equiv., 9: 1.8 equiv., 10: 2.0 equiv., 11: 2.2 equiv., and 12: 2.4 equiv.

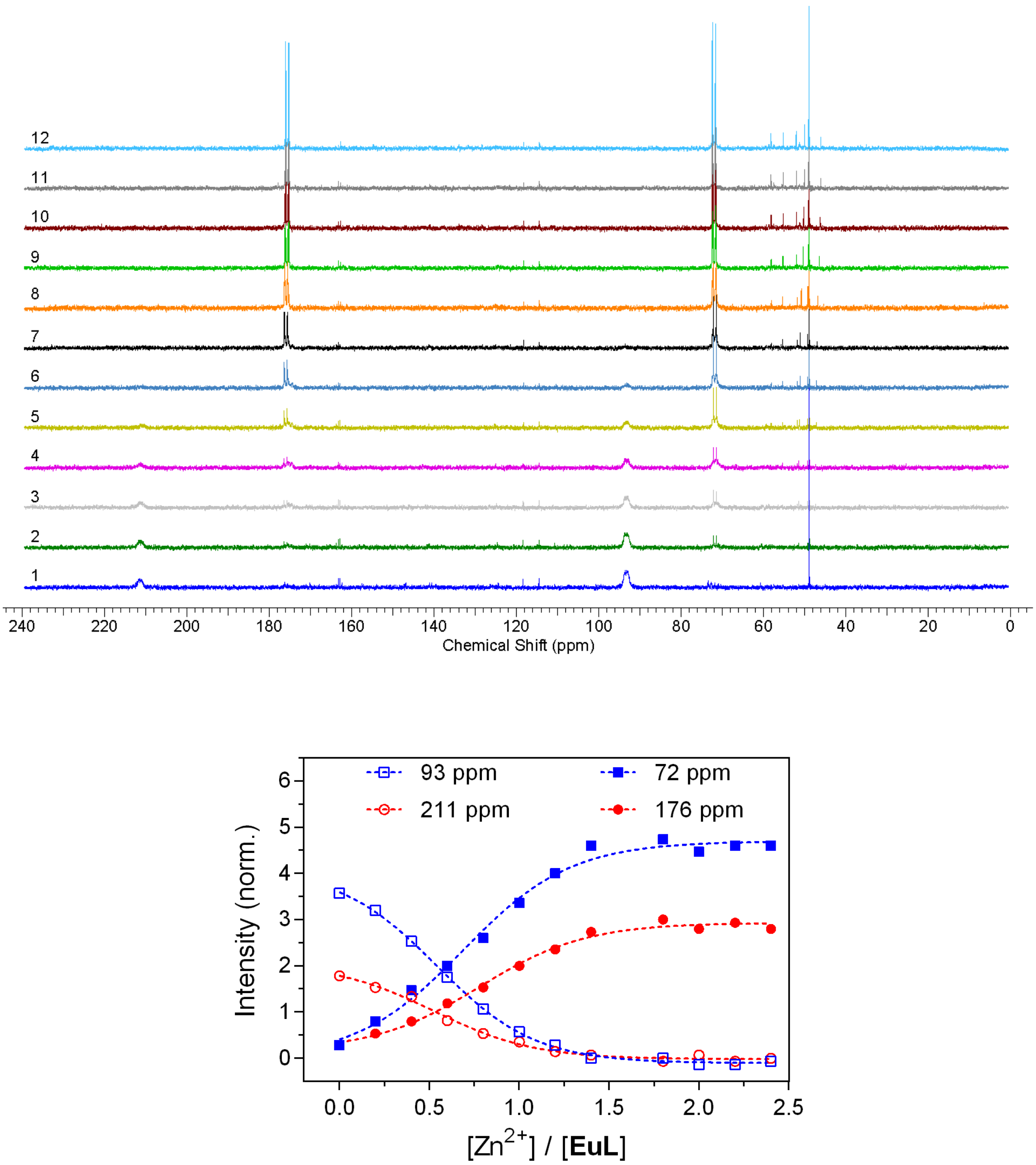

Figure S4. ^{13}C NMR spectra of **EuL*** (15 mM) in the presence of 0 to 2.4 equiv of Zn^{2+} (top) and the observed changes in ^{13}C NMR shifts with the additions of Zn^{2+} (bottom).

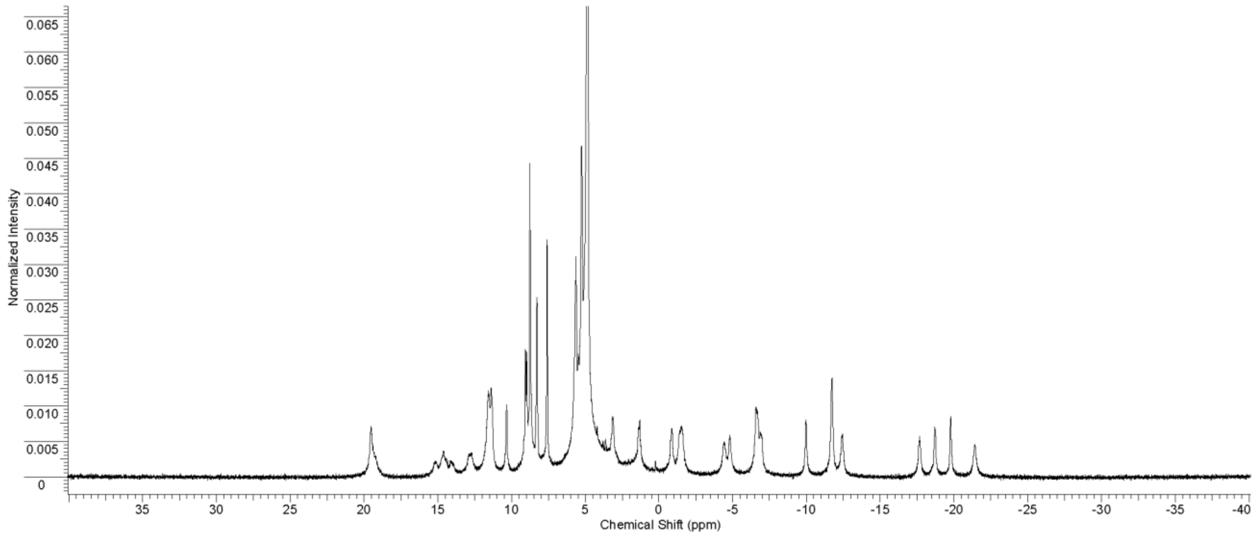

Figure S5. ^{1}H NMR spectrum of **EuL*** in D_2O at 25 °C.

Luminescence lifetime experiments

Luminescence lifetime measurements were performed with **TbL** (1 mM) or **EuL*** (5 mM) in D_2O and H_2O with and without Zn^{2+} (2 equiv.) at 25 °C (50 mM HEPES, pH 7.4). The Ln^{3+} ion was directly excited (λ_{ex} = 283 and 395 nm for Tb^{3+} and Eu^{3+}, respectively) and the emission intensity (λ_{max} = 546 and 615 nm for Tb^{3+} and Eu^{3+}, respectively) was recorded. The excitation and emission slits were set at 10 nm. In total, three independent measurements each with 15 scans were performed to obtain the data set. The obtained curves were fitted with a first order exponential decay with an $r^2 > 0.99$. The resulting q value, which denotes the hydration number of coordinated water molecules, was then calculated using the equation Eq. S2 for **EuL*** and Eq. S3 for **TbL**.[3]

$$q(\mathrm{Eu}) = 1.2 \times \left(\frac{1}{\tau_{\mathrm{H_2O}}} - \frac{1}{\tau_{\mathrm{D_2O}}} - 0.25\right) \qquad \text{Eq. S2}$$

$$q(\mathrm{Tb}) = 5 \times \left(\frac{1}{\tau_{\mathrm{H_2O}}} - \frac{1}{\tau_{\mathrm{D_2O}}} - 0.06\right) \qquad \text{Eq. S3}$$

MRI experiments

Tube phantoms studies: MRI phantoms were obtained using 6 x 400 μL vials, immersed in HEPES (50 mM, pH 7.4) solution. Apart from the first vial containing only 1 mM buffered (50 mM HEPES, pH 7.4) solution of the **GdL** complex, the rest vials were treated with $MgCl_2$ (1.0 equivalent), $CaCl_2$ (1.0 equivalent) and $ZnCl_2$ (0.5, 1.0, 2.0 equivqlents). The following parameters were used for MRI acquisition: FOV = 2.85 × 2.85 cm, MTX = 190 × 190, spatial resolution = 150 x 150 um, slice thickness = 0.5 mm, FA = 90°, TR = 20.0 ms, TE = 2.35 ms, TA= 3 m 10 s.

Table S1. Obtained SNR (signal-to-noise ratio) in T_1-weighted MRI of tube phantoms of different solutions with 1 mM of **GdL** (50 mM HEPES buffer**,** pH 7.4 and 298 K).

Sample	**GdL**	**GdL** + 1.0 eq Mg^{2+}	**GdL** + 1.0 eq Ca^{2+}
SNR	54.0	56.3	61.0
Sample	**GdL** + 0.5 eq Zn^{2+}	**GdL** + 1.0 eq Zn^{2+}	**GdL** + 2.0 eq Zn^{2+}
SNR	92.7	124.3	132.8

References

[1] G. Wang, C. Platas-Iglesias, G. Angelovski, *ChemPlusChem* **2020**, *85*, 806-814.

[2] D. M. Corsi, C. Platas-Iglesias, H. van Bekkum, J. A. Peters, *Magn. Reson. Chem.* **2001**, *39*, 723-726.

[3] A. Beeby, I. M. Clarkson, R. S. Dickins, S. Faulkner, D. Parker, L. Royle, A. S. de Sousa, J. A. G. Williams, M. Woods, *J. Chem. Soc., Perkin Trans. 2* **1999**, 493-503.
